# Field-based body temperatures reveal behavioral thermoregulation strategies of the Atlantic marsh fiddler crab *Minuca pugnax*

**DOI:** 10.1371/journal.pone.0244458

**Published:** 2021-01-06

**Authors:** Sarah Hews, Zahkeyah Allen, Adrienne Baxter, Jacquline Rich, Zahida Sheikh, Kayla Taylor, Jenny Wu, Heidi Zakoul, Renae Brodie

**Affiliations:** 1 School of Natural Science, Hampshire College, Amherst, MA, United States of America; 2 Department of Mathematics & Statistics, Amherst College, Amherst, MA, United States of America; 3 Biological Sciences, Mount Holyoke College, South Hadley, MA, United States of America; KAUST University, SAUDI ARABIA

## Abstract

Behavioral thermoregulation is an important defense against the negative impacts of climate change for ectotherms. In this study we examined the use of burrows by a common intertidal crab, *Minuca pugnax*, to control body temperature. To understand how body temperatures respond to changes in the surface temperature and explore how efficiently crabs exploit the cooling potential of burrows to thermoregulate, we measured body, surface, and burrow temperatures during low tide on Sapelo Island, GA in March, May, August, and September of 2019. We found that an increase in 1°C in the surface temperature led to a 0.70-0.71°C increase in body temperature for females and an increase in 0.75-0.77°C in body temperature for males. Body temperatures of small females were 0.3°C warmer than large females for the same surface temperature. Female crabs used burrows more efficiently for thermoregulation compared to the males. Specifically, an increase of 1°C in the cooling capacity (the difference between the burrow temperature and the surface temperature) led to an increase of 0.42-0.50°C for females and 0.34-0.35°C for males in the thermoregulation capacity (the difference between body temperature and surface temperature). The body temperature that crabs began to use burrows to thermoregulate was estimated to be around 24°C, which is far below the critical body temperatures that could lead to death. Many crabs experience body temperatures of 24°C early in the reproductive season, several months before the hottest days of the year. Because the use of burrows involves fitness trade-offs, these results suggest that warming temperatures could begin to impact crabs far earlier in the year than expected.

## Introduction

Warmer than average days and heat waves are becoming more frequent with climate change [1]. For ectothermic organisms that thermoregulate to keep their body temperatures close to a thermal optimum, *T*_*opt*_ [2–4], the physiological responses to elevated environmental temperatures are understood. Excessively warm days exert deleterious impacts on energy and water budgets [5–7], and more extreme environmental temperatures lead to anaerobic respiration, compromising cellular function [8–10]. Deviations from *T*_*opt*_, including those that surpass critical thermal limits, *CT*_*max*_, for short periods, can be survivable because organisms have compensatory mechanisms. For example, at a cellular level organisms can up-regulate genes associated with repair processes [11–13], and they can engage in behaviors that increase tissue oxygenation once a crisis has passed [14]. However, compared to endotherms, ectotherms have less control over internal processes that can generate or redistribute heat within their bodies and rely heavily on behavioral mechanisms to regulate body temperatures [15, 16]. Under the likely scenario of continued warming, behavioral thermoregulation will be a critical means for ectotherms to deal with high daily averages and avoid extreme temperatures, especially in locations like the southeastern United States that are already experiencing unusually high numbers of heat wave days [17]. For this reason, the efficiency with which ectotherms can use the surrounding environment to adjust their body temperatures and the cost associated with these behaviors are important determinants of vulnerability.

As a highly active intertidal forager, the fiddler crab is an excellent model system in which to investigate the impacts of environmental temperatures on thermoregulatory behaviors. Fiddler crabs remain in their burrows when tidal flats are flooded, but emerge during daytime low tides, when droves of foraging individuals roam exposed mud and sand flats to deposit feed on microflora and fauna [16, 18, 19]. Juvenile and adult fiddler crabs are preyed on by fishes, birds, and other crabs [20], while the planktonic larvae are consumed primarily by fishes, especially estuarine larval fishes [21]. Fiddler crabs, like other ectotherms, employ a variety of physiological, morphological, and behavioral strategies to thermoregulate during these periods of exposure. Evaporative cooling from a wetted body is possible on windy and less humid days [22, 23], and some species can readily change the distribution of chromatophores on their cuticles to increase reflectance [24, 25]. On hot days, fiddler crabs orient their bodies to minimize the surface area experiencing direct exposure from the sun [23] and males radiate heat from their enlarged claw [26] which is also an ornament and weapon used in courtship contests [27, 28]. As mobile ectotherms, they have the option to retreat to cooler microhabitats, including shade [23, 29], and especially the burrow [22, 23, 29].

For fiddler crabs, offloading heat in a burrow that is cooler than the surface is a highly effective thermoregulatory strategy [23, 30]. Burrow temperatures decline exponentially with depth and burrows maintain a more stable temperature profile compared to the surface [22]. Semi-permanent burrows extending 10-60 cm [31–33] into the substratum can exceed densities of 100 burrows/m2 in areas occupied by fiddler crab colonies (e.g., [34, 35]). Individual crabs can claim a burrow, modify it, and use it as a place to court, mate, incubate embryos [32], hide from predators [36, 37] and thermoregulate. Individuals that are foraging rather than defending a burrow are rarely more than a few body lengths from a burrow and can access unoccupied or poorly defended burrows as they travel across the substratum (Brodie, pers. observ.). The cooling capacity and abundance of burrows may explain why researchers report finding fiddler crabs active when surface temperatures exceed their critical thermal limits [29, 38] (Brodie, pers. observ.).

It is now recognized that ectotherm survival in warming habitats may depend on their ability to exploit microclimates to manage body temperatures [39, 40]. Here, we used the Atlantic marsh fiddler crab *Minuca pugnax*, a temperate species with a range from New Hampshire to northern Florida USA [41], as a model system to investigate relationships between body temperature, *T*_*b*_, surface temperature, and burrow sediment temperatures. *T*_*b*_ is influenced by heat exchange between the organism and the environment through radiation from the sun and other objects, as well as convection and conduction [42]. We measured sediment temperatures because fiddler crabs, like many other errant marine intertidal invertebrates, have wet bodies that are in nearly constant contact with wet sediment, making thermal flux between the surface, burrow and body an important determinant of *T*_*b*_. We explored the usefulness and limits of burrows for thermoregulation. In this study, we employed several parameters that are commonly used in thermal ecology research, and introduced three new parameters:

- $\hat{T_{e}}$, the operative body temperature when the burrows offer no thermal refuge (*T*_*e*_ is usually used for the operative body temperature [43–45]);
- *T*_*reg*_, the lowest body temperature at which an ectotherm starts to behaviorally thermoregulate using the burrows; and
- *E*_*B*_, the burrow use efficiency, or how efficiently the crabs use the burrows to thermoregulate.

Specifically, we asked: (1) How are *T*_*b*_ values impacted by surface temperatures and what *T*_*b*_ values are experienced during the active season (spring, summer and fall) near the southern end of the species range; (2) How efficiently can crabs exploit the cooling potential of burrows to thermoregulate, and how do changes in the cooling potential of the burrows impact thermoregulation; and (3) At what *T*_*b*_ does a crab begin to use burrows to resist heat transfer from the environment (when does *T*_*b*_ = *T*_*reg*_)?

## Materials and methods

### Field based body temperatures (*T*_*b*_)

Two field sites were located on Sapelo Island, a 6677 hectare barrier island off the coast of Georgia, USA at Shell Hammock (31.399832,-81.287417), which included both high and low marsh, and Lighthouse Road (31.390724, -81.285953), a low marsh site. Collection sites and procedures were approved by the University of Georgia’s Marine Institute on Sapelo Island (University of Georgia field permit number #1000531205). At both sites, low tides exposed patches of mud and sand interspersed with plant cover, including *Spartina alterniflora*, *Distichlis spicata*, *Salicomia virginica*, *Sarcocornia perennis*, and *Juncus roemerianus*. While *Minuca pugnax* was the dominant fiddler crab species, *Leptuca pugilator* was also present. Sapelo Island experiences semidiurnal tides with an average range of 2.08 m (NOAA Tides and Currents), and aerial temperatures around the time of the study were 27-29°C in July and August, the hottest months of the year, with maximum temperatures reaching 7-9 degrees higher than these monthly averages [46].

*T*_*b*_ measurements of 441 male (*n* = 252) and female (*n* = 189) *M. pugnax* were collected during low tide in 2019 on 29 and 30 March; 10, 11 and 13 May; 24, 26 and 28 August, and 12 and 13 October. The body temperatures, *T*_*b*_, of surface active male and female *Minuca pugnax* were measured during the daytime low tides using a Physitemp Instruments portable temperature monitor (PTM1) with a Type T needle microprobe (MT-29). Individual crabs of around 1 cm carapace width or larger were picked up from the surface opportunistically and the needle probe was inserted between the 2nd and 3rd walking leg into the gill chamber for a temperature reading (increment: 0.1°C). Following the *T*_*b*_ measurement, the carapace width (CW) was measured with a digital caliper (0.1 mm increment) and the crab was released. The same microprobe was used to measure the surface temperature at the location where each individual crab was caught, and every 10-30 minutes during the collection period a temperature measurement was taken from the bottom of a nearby artificial burrow made from a 2.5 cm wide pvc pipe, extending 30 cm into the substratum. We could not use real burrows because their curves and inclines made it too difficult to thread in the microprobe and take measurements at a consistent depth. This reference depth was within the range that crabs can access and ensured that the measurement captured the coolest burrow microclimate available at that time and place, as burrow temperatures decline exponentially with depth, with most of that change occurring between the surface and a depth of 15 cm [47]. The artificial burrow was moved frequently to keep it close to areas where crabs were being measured. Burrow temperatures tended to remain stable over the course of the measurement period, indicating that the pvc pipe did not cause warming or impede cooling.

Males and females of the same carapace width are not comparable because of the male’s enlarged sexually selected claw. To enable us to calculate parameter values, in most of the statistical analyses we treated size as a dichotomous categorical variable divided at the midpoint in the range of sizes collected for each sex. Four classes of crabs were created: small females (9-12.9 mm CW), large females (13-17 mm CW), small males (10-14.9 mm CW), and large males (15-20 mm CW). We confirmed our conclusions by running statistical analysis using size as a continuous variable for each sex.

To determine if there were differences in *T*_*b*_ between the small and large females and the small and large males, we used a Kruskal-Wallis H test followed by pairwise comparisons with a Dunn’s (1964) procedure and a Bonferonni correction for multiple comparisons. An ANOVA was not used because the assumption of normality was not met after multiple transformations were tried. The four sampling months, March, May, August and October 2019 were analyzed separately.

Using the field based *T*_*b*_ and surface temperature, *S*, measurements described above, we calculated the difference between the body and surface temperature, *T*_*b*_ − *S*, for each crab, where a positive value indicated that the crab was warmer than the surface and a negative value indicated that it was cooler. Separate regressions for the four categories of crab, with surface temperature as the independent variable and *T*_*b*_ − *S* as the dependent variable, were plotted and used to investigate crab abilities to maintain body temperatures different from the surface, including the specific *T*_*b*_ at which crabs began to use burrows to cool themselves (the *T*_*reg*_ estimation described below). For each sex, we investigated the effect of size with an ANCOVA, where *T*_*b*_ − *S* was the continuous dependent variable, crab size was the categorical independent variable, and surface temperature was the continuous covariate. We tested for the homogeneity of regression slopes by investigating the interaction between surface temperature and crab type in the GLM. For females, untransformed values of the dependent variable were used and the model met assumptions of linearity, homogeneity of regression slopes, normality, homoscedasticity, homogeneity of variances, and there were no outliers. While most of the model assumptions were met for males, the residuals for large males were not normally distributed, the homogeneity of variances was violated, and there were four outliers. While a log10 transformation addressed the problem with homogeneity of variances and adjusted three of the four outliers, no transformation was found that could normalize the residuals. For this reason, we evaluated the impacts of male size and surface temperature on *T*_*b*_ − *S* with a Permutational Analysis of Variance (PERMANOVA), a non-parametric approach that fits linear models to distance matrices [48]. We ran 999 permutations to test model significance, using the Adonis function from the vegan package of R [49].

### Estimating the burrow use efficiency (*E*_*B*_) and the difference between the operative body temperature and the surface when burrows offer no thermal refuge ($\hat{T_{e}}-S$)

We introduced a new parameter, *E*_*B*_, a metric that describes how efficiently the crabs use burrows to thermoregulate. The burrow use efficiency, *E*_*B*_, was determined by finding the rate of change of the relationship between two newly defined terms: (1) the cooling capacity of the burrow as the potential of the burrow to cool the crab (*T*_*b*_ − *S*), and (2) the thermoregulation capacity as the ability of the crab to use the burrow, *B*, and other means to regulate body temperature (*B* − *S*). Theoretically, the burrow could also be used to heat the crab when the burrow is warmer than the surface during the night and early morning. The greater the difference between crab and surface temperature, the larger the crab’s thermoregulation capacity or the degree to which it was managing its body temperature through behavioral, physiological, and morphological mechanisms.

The cooling capacity of the burrow, represented by the *x* variable, is the difference between the burrow temperature and the surface temperature, *x* = *B* − *S*. The behavioral thermoregulation capacity of the crab, represented by the *y* variable, is the difference between the crab body temperature and the surface temperature, *y* = *T*_*b*_ − *S*.

**Fig 1:**
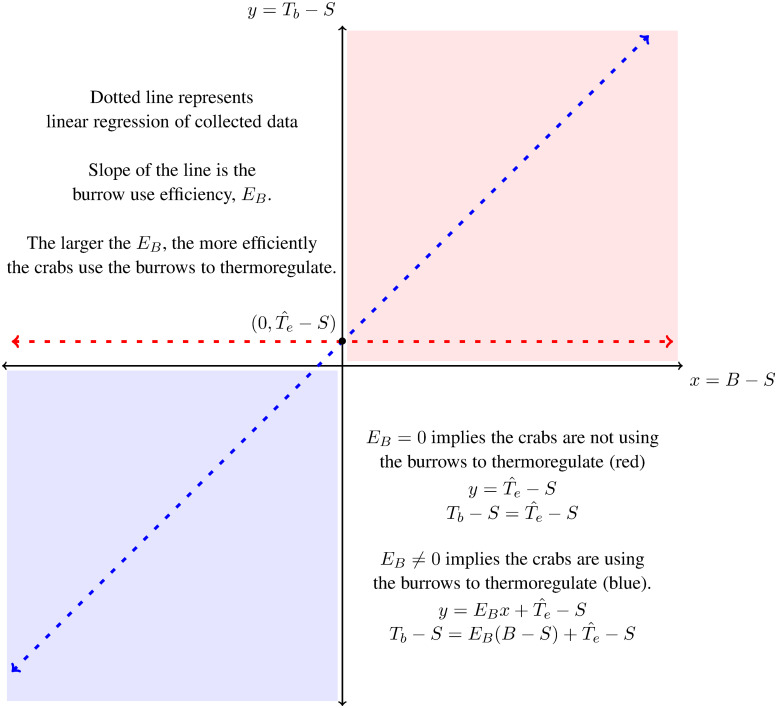
The thermoregulation axis. The graph of the thermoregulation capacity, *T*_*b*_ − *S*, against the cooling capacity, *B* − *S* of the burrow. The slope of the dotted line, that represents the linear regression of the collected data, is the burrow use efficiency, *E*_*B*_.

We represent the relationship between the cooling/heating capacity of the burrow and the behavioral thermoregulation capacity of the crab in a coordinate plane (Fig 1). We call this representation of the relationship the thermoregulation axes. The blue thermoregulatory zone corresponds to *x*, *y* < 0, where both the burrow and the crab are cooler than the surface. Theoretically, this zone represents the crab using the burrow to thermoregulate when the surface is too warm. The red thermoregulatory zone corresponds to *x*, *y* > 0, where the burrow and the crab are warmer than the surface, and represents the crab using the burrow to warm itself through thermoregulation when the surface is too cold.

In the scenario where the crabs do not use the burrow to thermoregulate, then data collected of crab, burrow, and surface temperature would fall along a horizontal line (Fig 1). If the crabs use the burrow to completely regulate their temperature, then data collected of crab, burrow, and surface temperature would fall along a line with slope 1 (Fig 1). Both scenarios would have data with a positive y-intercept that corresponds to the difference between the surface temperature and the crab body temperature. When burrows are used to thermoregulate, the y-intercept marks the point where no thermoregulation with the burrow is occurring because burrow and surface temperatures are the same. We expect the y-intercept to be greater than zero because the thermal properties of their bodies cause crabs to be warmer than the surface when they are not thermoregulating. On a sunny day in August, 2019, we found that dead crabs and crabs filled with silicone (also dead) on an unshaded area of the marsh were 0.5-2.0°C warmer than the surface after 30 min (unpublished data for males).

The crab, burrow, and surface temperature data therefore falls on a line *y* = *E*_*B*_ *x* + *b* on the thermoregulation axes where *E*_*B*_ is defined as the burrow use efficiency and *b* is how much warmer crabs are than the surface and set by material properties of the organism. *b* can also provide another estimate for the difference between the operative body temperature when burrows offer no thermal refuge, $\hat{T_{e}}$, and the surface temperature, $S:b=\hat{T_{e}}-S$.

The relationship between the cooling/heating capacity of the burrow and the thermoregulation capacity (the thermoregulation axes) were investigated for females using an ANCOVA analysis, where the thermoregulation capacity of the crab (*T*_*b*_ − *S*) was the continuous dependent variable, crab size (large and small) was the categorical independent variable, and the cooling capacity of the burrow (*B* − *S*) was the continuous covariate. A square root transformation of the dependent variable was used for females to meet the assumption of normality. The residuals for large males were not normally distributed and this could not be fixed with transformations. For this reason, we used the non-parametric PERMANOVA [48] to evaluate the impacts of size and the cooling capacity of the burrow on male thermoregulation capacity. We ran 999 permutations to test model significance, using the Adonis function from the vegan package of R [49].

### Estimating the temperature that the crab begins to thermoregulate (*T*_*reg*_) due to the burrow

**Fig 2:**
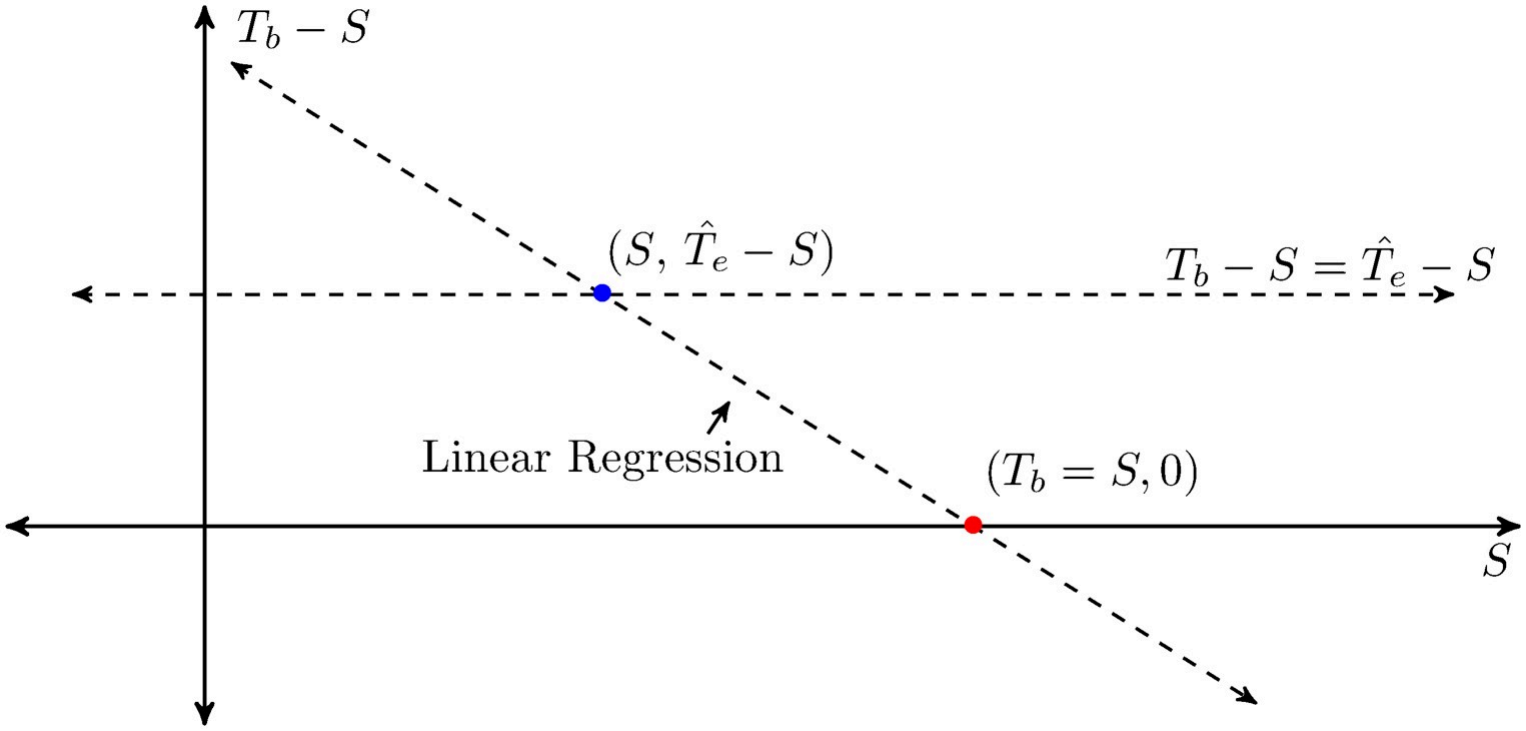
*T*_*b*_ is the crab body temperature, *S* is the surface temperature, *B* is the burrow temperature, and $\hat{T_{e}}$ is the operative body temperature when burrows offer no thermal refuge (*B* = *S*). The x-coordinate of the linear regression of *T*_*b*_ − *S* vs. *S* (red point) provides an initial estimate for *T*_*reg*_. A more precise *T*_*reg*_ is found by finding the surface temperature where $T_{b}-S=\hat{T_{e}}-S$ (blue point) and then adding $\hat{T_{e}}-S$ to get the corresponding crab body temperature.

An estimate for *T*_*reg*_, the body temperature that the crab begins to thermoregulate using the burrow, can be calculated by using the linear regression of the graph *T*_*b*_ − *S* vs. *S* (Fig 2). The x-intercept (red dot) provides an upper bound estimate of *T*_*reg*_ but a more precise estimate is found by using the operative body temperature when the burrows offer no thermal refuge, $\hat{T_{e}}$ to get an estimate for how much warmer crab *T*_*b*_ is than the surface due to crab thermal properties (e.g, carapace color). This enables us to find the surface temperature when $T_{b}-S=\hat{T_{e}}-S$. After finding the *S* value where $\hat{T_{e}}-S=T_{b}-S$, we added $\hat{T_{e}}-S$ to the surface temperature to find the corresponding body temperature above which the crab must be thermoregulating.

## Results

### Field based body temperatures (*T*_*b*_) during the active season, and the influence of size and surface temperature on thermoregulation capacity (*T*_*b*_ − *S*)

**Fig 3:**
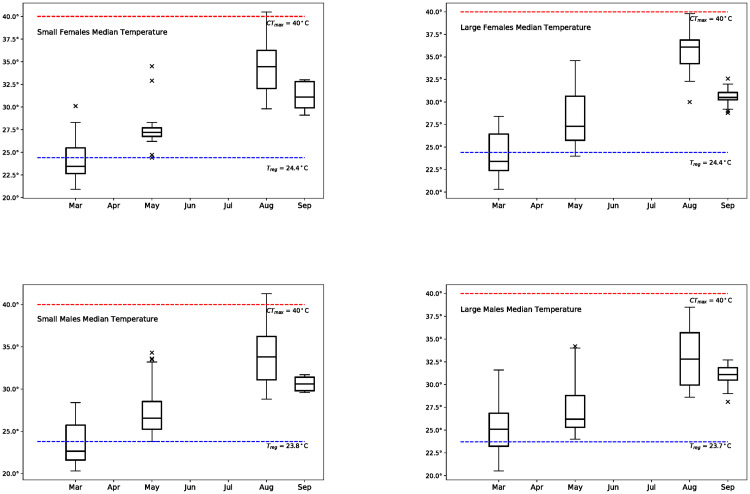
Box plots showing *T*_*b*_ for small females, large females, small males, and large males for March, May, August, and September. Red dashed line represents *CT*_*max*_ = 40° *C*, the critical thermal temperature, or the maximum *T*_*b*_ at which performance is possible. For fiddler crabs this has been measured as the *T*_*b*_ at which they lose their righting response [29, 50]. The blue dashed line represents estimates of *T*_*reg*_ (calculated later in this article).

Median *T*_*b*_ values in March were 23.5°C (*n* = 22; small females), 23.4°C (*n* = 19; large females), 22.7°C (*n* = 38; small males), and 25.1°C (*n* = 38; large males) (Fig 3). There was an overall significant difference among *T*_*b*_ distributions in March (*H*(3) = 7.89, *p* = 0.048), where small and large males showed significantly different distributions from each other (Fig 3). In May, median *T*_*b*_s were 27.2°C (*n* = 19; small females), 27.3°C (*n* = 51; large females), 26.6°C (*n* = 48; small males) and 26.2°C (*n* = 29; large males) and there were no significant differences in *T*_*b*_ across groups (*H*(3) = 4.08, *p* = 0.253; Fig 3). Median *T*_*b*_ values were highest in August at 34.5°C (*n* = 24; small females), 36.1°C (*n* = 22; large females), 33.8°C (*n* = 32; small males), and 32.8°C (*n* = 35; large males). There was an overall significant difference in *T*_*b*_ distributions in August (*H*(3) = 11.43, *p* = 0.01), with a significant difference between large males and large females (*p* = 0.006; Fig 3). In October, median *T*_*b*_ values were 31.3°C (*n* = 5; small females), 30.5°C (*n* = 27; large females), 30.6°C (*n* = 9; small males), and 31.1°C (*n* = 23; large males). No significant differences in *T*_*b*_ distributions were found for October (*H*(3) = 2.97, *p* = 0.413; Fig 3); however, the low sample sizes in two of the groups likely resulted in a low statistical power for this analysis.

**Fig 4:**
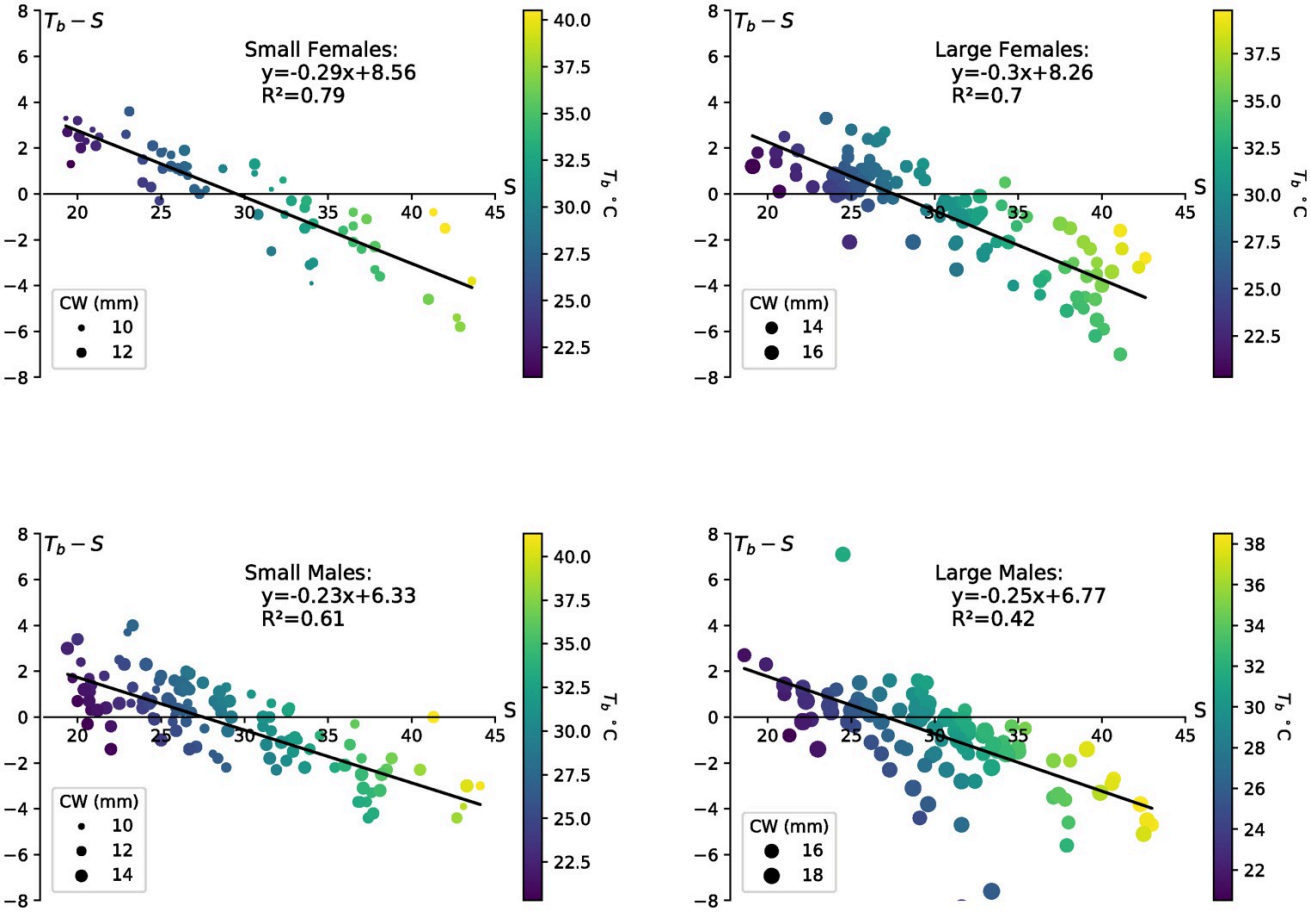
*T*_*b*_ − *S* against *S* and linear regressions for small females, large females, small males, and large males. The color of the data point corresponds to the body temperature on the color bar and the size of the data point represents the carapace width.

Of 441 *T*_*b*_ measurements taken, we also collected surface and burrow temperatures for 220 males and 157 females. While surface temperatures were checked immediately following each body temperature measurement, artificial burrow temperatures were checked less frequently, at 16 minute intervals during the data collection period on average. The artificial burrow temperatures fluctuated a half degree (*M* = 0.5, *SD* = 0.57; *n* = 24) between these readings compared to the surface which showed greater temperature variation for the same intervals (M = 1.4, SD = 1.7; n = 24). The relationship between the thermoregulation capacity, *T*_*b*_ − *S*, and the surface temperature, *S*, was determined to be linear for females and small males, with 79% (small females), 70% (large females), 61% (small males), and 42% (large males) of the thermoregulation capacity explained by the surface temperature (Fig 4). For females, both the surface temperature (*F*_1,154_ = 454.2, *p* < 0.0005) and crab carapace width (*F*_1,154_ = 7.8, *p* = 0.006) were significant predictors of *T*_*b*_ − *S* and there were no interaction effects (*F*_1,154_ = .146, *p* = .703). The interpretation of these results is that for every 1°C increase in surface temperature, the thermoregulation capacity of females decreased by approximately 0.29°C–0.30°C and for every increase in surface temperature of 1°C, *T*_*b*_ in females increased by 0.70°C-0.71°C. The significance of carapace width implies that the *T*_*b*_ of small females was 0.3°C warmer than large females for the same surface temperature (Fig 4). These results were congruent when carapace width is treated as a continuous variable.

For males, both the surface temperature (*F*_*pseudo*_ = 107.2, *p* = 0.001) and carapace width (*F*_*pseudo*_ = 4.0, *p* = 0.015) were significant predictors of *T*_*b*_ − *S* and there were no interaction effects (*F*_*pseudo*_ = 1.8, *p* = 0.137). The interpretation of the individual regressions for males is that for every 1°C increase in surface temperature, the thermoregulation capacity of males decreased by approximately 0.23°C -0.25°C and for every increase in surface temperature of 1°C, the *T*_*b*_ for males increased by 0.75°C-0.77°C. (Fig 4).

### Estimating the burrow use efficiency (*E*_*B*_) and the difference between the operative body temperature and the surface when burrows offer no thermal refuge ($\hat{T_{e}}-S$)

**Fig 5:**
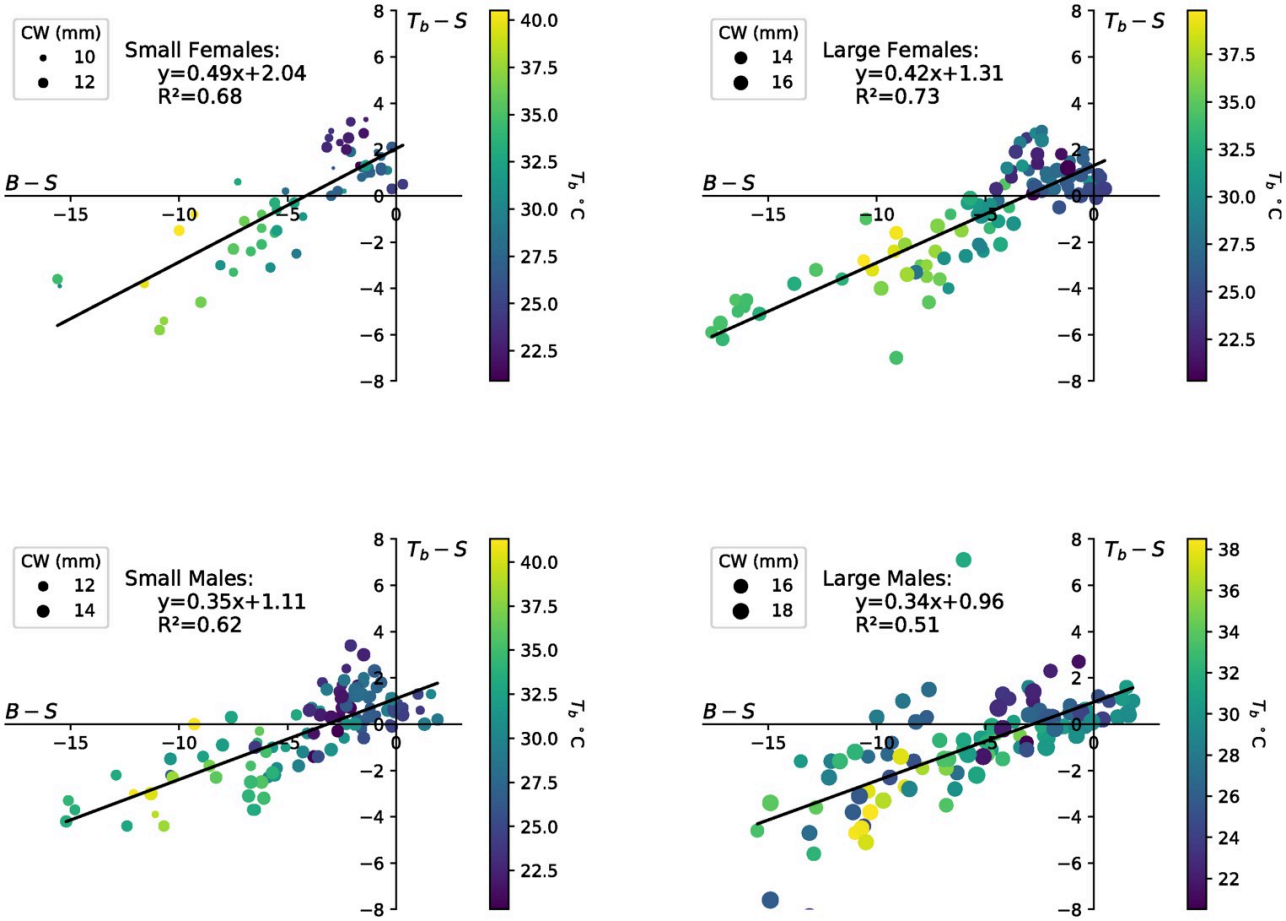
*T*_*b*_ − *S* against *B* − *S* and linear regressions for small females, large females, small males, and large males. The color of the data point corresponds to the body temperature on the color bar and the size of the data point represents the carapace width.

The relationship between the thermoregulation capacity, *T*_*b*_ − *S*, and the cooling capacity of the burrow, *B* − *S*, was determined to be linear for females and small males, with 68% (small females), 73% (large females), 61% (small males), and 50% (large males) of the thermoregulation capacity explained by the cooling capacity of the burrow (Fig 5).

For females, the cooling capacity (*F*_1,154_ = 330.6, *p* < 0.0005) was a significant predictor and the crab carapace width (*F*_1,154_ = 3.8, *p* = 0.054) tended towards significance as a predictor of the thermoregulation capacity. There were no interaction effects (*F*_1,154_ = 3.4, *p* = .069). This implies that the burrow use efficiency (*E*_*B*_) was not significantly different between small females (*E*_*B*_ = 0.5) and large females (*E*_*B*_ = 0.42), meaning that there was no significant difference in how thermoregulation capacity changes in response to changes in the cooling capacity of the burrow. For each 1°C increase in the cooling capacity (meaning *B* − *S* decreases by 1°), females increased their thermoregulation capacity by 0.42°C-0.50°C (meaning *T*_*b*_ − *S* decreased by 0.42°C-0.50°C). For a set cooling capacity, the thermoregulation capacity was 0.7°C greater for large females than small females. Alternatively, large females reduced their temperature by 0.7°C more than small females for a given cooling capacity and the same environmental conditions (same *B* and *S*). The y-intercept was 2.04°C for small females and 1.31°C for large females. This means that when the burrow offered no thermal refuge, *B* = *S*, $T_{b}-S=\hat{T_{e}}-S$ was 2.04°C for small females and 1.31°C for large females (Fig 5). These results were congruent when carapace width is treated as a continuous variable.

For males, the cooling capacity (*F*_*pseudo* = 285.4_, *p* = 0.001) was a significant predictor of the thermoregulation capacity of the crab, while carapace width (*F*_*pseudo*_ = 1.1, *p* = 0.31) and the interaction between carapace width and cooling capacity (*F*_*pseudo*_ = 1.6, *p* = 0.22) were not significant predictors when carapace width was treated as a dichotomous categorical variable (large vs. small). However, when carapace width was treated as a continuous variable within the model, in addition to the cooling capacity, both carapace width (*F*_*pseudo*_ = 4.6, *p* = 0.026) and the interaction between carapace width and cooling capacity (*F*_*pseudo*_ = 5.8, *p* = 0.018) were significant predictors of the thermoregulation capacity, with larger males appearing to harvest (or retain) more of the cooling capacity than smaller ones. For each 1°C increase in the cooling capacity (meaning *B* − *S* decreases by 1°C), males increased their thermoregulation capacity by 0.34°C-0.35°C (meaning *T*_*b*_ − *S* decreased by 0.34°C-0.35°C). When the burrow offered no thermal refuge, *B* = *S*, $T_{b}-S=\hat{T_{e}}-S$ was between 0.96°C-1.11°C (Fig 5).

### Estimating the temperature that the crab begins to thermoregulate (*T*_*reg*_) due to the burrow

Using the estimates for $\hat{T_{e}}-S$ (2.04°C, small females; 1.31°C, large females; 1.11°C, small males; 0.96°C, large males) (Fig 5), and the regression lines establishing the linear relationship between *T*_*b*_ − *S* and *S* (Fig 4), *T*_*reg*_ was calculated as 24.52°C (small females), 24.48°C (large females), 23.81°C (small males), and 24.20°C (large males).

## Discussion

Ectotherms, like *Minuca pugnax*, will experience increased maintenance costs as air, water, and surface temperatures warm with climate change, requiring compensatory responses that include behavioral thermoregulation [40]. The critical thermal body temperatures, *CT*_*max*_, of ten different fiddler crab species have been found to lie between 40–43°C [38, 50–53], with *M. pugnax* showing a *CT*_*max*_ of 40° C [51]. These studies assessed thermal limits with the righting response, a procedure that can deliver different results depending on experimental conditions [54] and might underestimate *CT*_*max*_ [55]. Nevertheless, these estimates fall within the typical range for ectotherms, which across taxa, habitats, and latitudes, experience severe heat stress at *T*_*b*_ above 40°C with some exceptions [39, 56–58]. The median daytime *T*_*b*_ for *M. pugnax* was well below this during our August data collection period at 33°C and 36°C for large males and large females, respectively. However, 29 out of 113 crabs measured over three days were found on sediment that was 40°C or hotter, and seven of these individuals had a *T*_*b*_ that was 40°C or higher. All of these individuals were active and appeared healthy, although ostensibly at or close to the *CT*_*max*_ estimate for this species. Acclimation, or hardening, following exposure to high temperatures [59] during the summer months might explain this. Although we have no information on the frequency or duration of these periods of very high *T*_*b*_ and their impact on crab health and survival, it is possible that the median *T*_*b*_ and the frequency of very hot crabs would have been even higher if we had collected data during the hottest month of the year (July).

The linear relationships between thermoregulation capacity, *T*_*b*_ − *S*, and surface temperature, *S*, that we found for females of all sizes and for small males allowed us to explore the impact of warming surfaces on crab bodies. For females, we found that for every 1°C of surface warming, their bodies warmed about 0.7°C, a finding that quantifies the degree to which they resisted heat transfer from the surface. Quantifying changes in behavior, especially with respect to use of burrows and foraging locations, along with an energy budget analysis, could help to elucidate the costs of this resistance. Females in both size categories were large enough to mate and carry broods, so we did not expect behavioral differences related to reproductive behaviors or embryo incubation to impact their responses to surface warming. Larger females tended to be around a third of a degree cooler than smaller ones on surfaces of the same temperature, a difference that might have been due to a higher thermal inertia or longer legs lifting them higher above the substratum. The bodies of small males warmed about 0.77° C for every 1° C of surface warming. While our PERMANOVA analysis revealed that crab size had a significant impact on the difference between body and surface temperature, we could not directly compare the linear regressions of *T*_*b*_ − *S* vs. the surface temperature for small and large males because this relationship was clearly non-linear for the larger males.

Large males were in a class of their own, with far less of the variation in thermoregulation capacity explained by surface temperature compared to females and small males. There was a non-linear relationship between thermoregulation capacity and surface temperature, with some large males much cooler relative to the surface than expected for a linear relationship. It is not surprising that the relationship between thermoregulation capacity and surface temperature would be different for this group. Male fiddler crabs experience allometric growth during development, with the large claw becoming disproportionately larger as the crab grows [60–62], and this sexually selected appendage is known to also function as a heat sink that lowers core body temperatures [26]. Furthermore, claw and body sizes influence abilities to attract mates and defend burrows [37, 63, 64], and courtship behaviors impact thermoregulation. Males that are actively courting females may experience high body temperatures if they are displaying in open habitats where they can be seen or higher in the intertidal where females prefer the more stable burrows [53, 65]. However, courting males also crowd into shaded areas that give them a thermal advantage [29, 66, 67], and those actively defending burrows have ready access to a cool microclimate. Hence, individual courting males may experience higher or lower than expected *T*_*b*_ depending on local conditions, including the availability of shade, female habitat preferences, and variation in the intensity of sexual competition. Because we collected crabs opportunistically, we probably collected some large males that were actively courting and some that were not. Although it is not clear to us whether courting or non-courting males would have been expected to have cooler *T*_*b*_ values at the same surface temperatures, sorting large males into different behavior categories might have elucidated the relationship between their thermoregulation capacity and surface temperature. Alternatively, this relationship might simply be inherently more variable and nonlinear for this group.

Fiddler crab habitats are riddled with burrows that extend tens of centimeters into the substratum, providing refuge from predators and inhospitable surface temperatures. Males entice females into burrows for matings and some species brood in them. The amount of time spent in burrows is influenced by season and local environmental conditions. During the daytime low tide exposure period, *M. panacea* spends around a quarter of its time in a burrow during the non-breeding season but nearly half of its time there while breeding [30]. The fiddler crab *Austruca mjoebergi* is almost as likely to be in its burrow as it is feeding on the surface when its burrow is in the sun, but time in the burrow drops dramatically in shade, where it is more than three times as likely to be feeding on the surface [67]. The fact that crabs spend less time in burrows when those burrows are shaded suggests a costly tradeoff between feeding, courtship, and thermoregulation, costs that may increase for *M. pugnax* during the reproductive season when it experiences declines in fat stores [68].

During 2019 on Sapelo Island, Georgia, USA burrows offered an important, if potentially costly, thermal refuge for *M. pugnax*. We found that most of the thermoregulation capacity for females was explained by the cooling capacity of the burrow–as the burrow became cooler relative to the surface, so did female *T*_*b*_. This was especially true for large females where over 70% of their thermoregulation capacity was explained by the cooling capacity of the burrow. We found that for a given cooling capacity, larger females were cooler than smaller ones when burrow and surface temperatures were held constant, and large females tended to be cooler than small ones when the burrow could not be used for thermoregulation (i.e., when burrow and surface temperatures were the same). These consistent findings could be explained by thermal inertia and the longer legs of large females. We estimated that for every 1.0 °C degree cooler the burrow became relative to the surface, large females could cool their bodies down an additional 0.5°C.

For males, our PERMANOVA results showed that cooling capacity was a strong predictor of thermoregulation capacity, regardless of whether crab size was treated as a binary or continuous trait. However, crab size was itself a significant predictor of thermoregulation capacity when it was treated as a continuous trait, but not a categorical one. This is the only analysis that we did for which this was the case. As burrows become cooler relative to the surface, it appears that the largest males take advantage of this cooling capacity more efficiently than smaller males do and this shows up in the analysis where size is treated as a continuous variable. In the future, very large males warrant more investigation to determine if this difference is due to behavior, physical properties or both.

Compared to females, males clearly used the burrow less efficiently for thermoregulation, cooling their bodies around a third of a degree for every 1°C increase in the cooling capacity of the burrow. They were also only around a degree hotter than the surface when the burrow could not be used for thermoregulation, compared to small females which were 2°C hotter. Furthermore, less of the variation in thermoregulation capacity was explained by the cooling capacity of the burrow, with only about 50% of that variation explained for large males. Males appear to rely less on the burrow for thermoregulation than females, quite possibly because they are larger on average and their enlarged claw provides an additional means for cooling down. They are also less efficient feeders [69] and face selective pressure to remain on the surface to court females [70], which may lead to more time on the surface and a greater reliance on thermoregulatory mechanisms that don’t involve the burrow. This study was also limited by the indirect way in which we had to assess the impact of burrows on *T*_*b*_. When wearable temperature loggers become small and light enough for fiddler crabs, we will be able to measure that impact directly.

Our estimates of the burrow use efficiency, *E*_*B*_, are conservative because we used burrow temperatures at 30 cm in our calculations, a reference depth that captured the coolest burrow microclimate available. If crabs were actually retreating to shallower depths where temperatures were not as cool, then the cooling capacity would have been reduced and the burrow use efficiency, *E*_*B*_, estimate would have been higher. In future work, measurements of how deep crabs go and their duration at these depths could be used to refine estimates of *E*_*B*_. We also think that it would be important to compare the cooling capacity of burrows in different habitats and climates, as places with similar surface and air temperatures but different burrow cooling capacities would present dissimilar thermal landscapes. For example, it appears that *M. pugnax* in the southern United States experiences surface temperatures in the summer that are similar to those experienced by *Tubuca urvillei* in Kenya. However, the surface and 20 cm depth temperatures measured by [71] in Kenya were not very different, suggesting that the cooling capacities of burrows there may be low. If this is true, it would not be surprising to find that *T. urvillei’s* thermoregulation capacity is lower than that of *M. pugnax*, which would result in different evolutionary pressures and responses to climate warming for the two species.

Finally, we found that male and female crabs of different sizes showed different thermoregulation capacities, burrow use efficiencies, and *T*_*b*_ values when the burrow could not be used for thermoregulation. However, the *T*_*b*_ at which they all started using burrows to thermoregulate, *T*_*reg*_, was around of 24°C, suggesting that *T*_*reg*_ is a parameter that is set by *M. pugnax’s* physiology, regardless of sex and size. Because we used data collected from March through October, this *T*_*reg*_ estimate should be viewed as an average for the 2019 breeding season on Sapelo Island, Georgia. In future work, seasonal and latitudinal differences in *T*_*reg*_ could be investigated to determine the degree to which acclimation and local adaptation influence this parameter. We also think that questions about the relationships between *T*_*reg*_, *T*_*opt*_, and *T*_*set*_, the preferred body temperature chosen by an ectotherm on a thermal gradient [2, 72, 73], should be investigated. Is the *T*_*b*_ at which field-based crabs start to use burrows, *T*_*reg*_, closer to the preferred *T*_*b*_ of *M. pugnax* in the lab, *T*_*set*_, or to the optimum temperature, *T*_*opt*_, for some aspect of its performance? For other ectotherms, *T*_*opt*_ within the same species can vary for different activities, like feeding, digestion, and running [4], and *T*_*set*_ has often been found to be lower than *T*_*opt*_, possibly to avoid the steep fitness drops that occur when *T*_*opt*_ is exceeded [74]. In *M. pugnax*, *T*_*reg*_ could hew closely to the *T*_*opt*_ of a particular fitness trait, it might be a compromise between several traits, or it could be conservatively low to avoid fitness costs. If *T*_*reg*_ and *T*_*set*_ are different, determining whether crabs start to use burrows more frequently before or after *T*_*set*_ will elucidate tradeoffs and strategies as they respond to their warming environments.

## Conclusion

While very hot days at the peak of summer can push intertidal invertebrates close to or above their *CT*_*max*_, the deleterious impacts of warmer days earlier in the reproductive season are less obvious. For *M. pugnax*, changes in burrow use related to thermoregulation involve fitness trade offs because time spent in burrows results in lost feeding and courtship opportunities [36, 75, 76]. However, while using a burrow to thermoregulate is potentially costly, it is also highly effective. As the surface warms rapidly through the day during the reproductive season in temperate climates, burrow temperatures remain cool and stable. For example, during a 48 hour period in May, we found temperatures in artificial burrows that were nearly 20°C cooler than the surface in the mid-afternoon when surface temperatures were hottest (burrows were warmer than the surface from evening to late morning; unpublished data). We found that crabs used burrows to counteract *T*_*b*_ increases, and we estimated *T*_*reg*_, the *T*_*b*_ at which crabs began to use burrows for thermoregulation, to start at around 24°C for *M. pugnax*. In late March, we found that many crabs showed *T*_*b*_ at or above this temperature, and when we returned in May the median *T*_*b*_ for crabs of all sizes and both sexes were above *T*_*reg*_. This was still the case for our last collection in October. With climate warming, *M. pugnax* will experience higher metabolic rates like all ectotherms, but there will be additional energy budget impacts as crabs spend more time cooling down in burrows beginning earlier in the year.
